# Education and information to improve rates for attendance to colorectal cancer screening programs

**DOI:** 10.1016/j.sopen.2024.03.017

**Published:** 2024-03-31

**Authors:** Raimondo Gabriele, Monica Campagnol, Immacolata Iannone, Valeria Borrelli, Antonio V. Sterpetti

**Affiliations:** Department of Surgery, University of Rome Sapienza, Italy

**Keywords:** Colorectalcancer, Screening, Adherence to screening

Dear Editor

We read with much interest the paper by Ng et al [1] The Authors analyzed the National Inpatient Sample, which include almost 97 % of the hospitalization in USA, concerning the incidence of emergency surgery for colorectal cancer. The Authors analyzed 722,736 patients who had admissions for colorectal cancer in USA from 2011 to 2020. Non-White race was associated with increased odds of emergent colorectal cancer resection. Trend analysis revealed that non-White patients consistently comprised over 5 % greater risk-adjusted proportion over the study period. Medicaid and uninsured status were significantly associated with *Emergent* admissions compared to private insurance. The Authors correctly pointed out that one of the main reason for this discrepancy was related with reduced screening program adherence correlated with race. The Authors pointed out several reasons for this reduced screening related with race, including lack of health literacy, lack of prompt screening recommendations, and lack of financial capital, insurance coverage, and access to primary care. The Authors correctly concluded “systematic approaches to alleviate racial inequities in colorectal cancer screening and improve access to timely surgical treatment are warranted” (Fig.1).

**Fig. 1:**
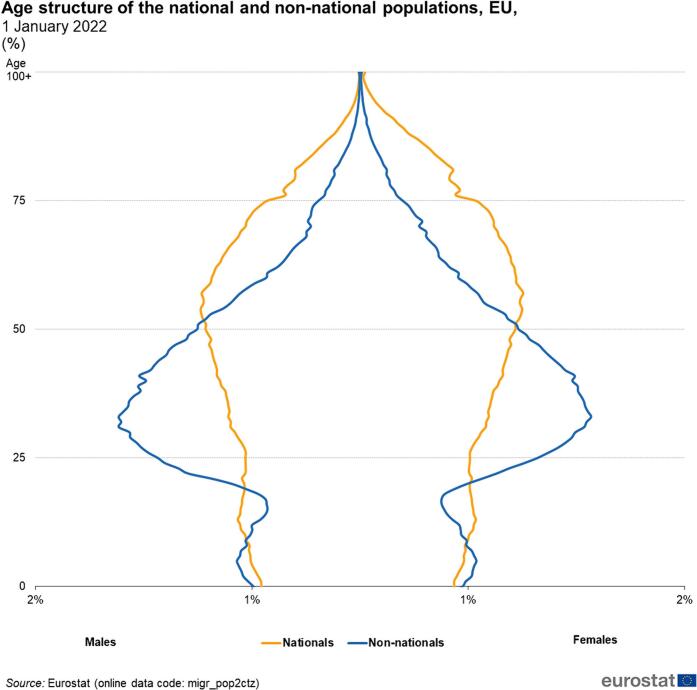
Age of national and immigrants from low-income countries in Italy and Western Europe.

The Authors used multiple regression analysis to determine risk factors correlated with emergent admissions for patients with colorectal cancer. Level of educational attainment and family income were not included in their analysis.

We agree completely with their conclusions, and we would like to add a specific consideration concerning educational attainment, information to the general population, and family income [[2], [3], [4]].

We reviewed the number of colorectal cancer screenings performed in Italy before and during the pre-pandemic years 2018 and 2019 [5]. The Italian Heath System allows free screening with Fecal Occult Blood Test for all the Italian population, including immigrants, aged 50–69 years. In Italy almost 4.5 % of the population includes non-white immigrants coming from low-income countries (North Africa, Asia, Central-South America) [6].

Invitation letters for screening were sent to an average of 6 million person per year (around 35 % of the Italian population aged 50–69 years). Half of the invited persons adhered to the screening; 5 % of the screened population had a positive test and underwent colonoscopy.

Univariate analysis showed a statistically significant lower rate of attendance to invitation for colorectal screening for people aged 50–59 years, people with lower educational attainment, and with lower family income. Immigrants had a lower rate of adherence without reaching a statistically significance (Table 1); however, we should consider that the percentage and number of non White persons in Italy is less than in USA.

**Table 1:** Screening for colorectal cancer (fecal occult blood test) in Italy.

	2018–2021	2020–2021
Colorectal cancer screening- fecal occult blood test		
Number invited	11.860.214	10.575.927
Number screened	5.190.308	4.094.965
% Positive test	5.0 %	5.3 %
N colonoscopies after positive test.	203.196	159.854
Diagnosis of carcinoma	5.295 1.2/1000 screening tests	4.080 0.94/1000 screening tests
Diagnosis of large adenoma	32.226 6.4/1000 of screening tests	26.306 6.5/1000 screening tests

Attendance to screening programs		
Age 50–59 Age 60–69	43 % 53 %	40 % 49 %
Females Males	47 % 49 %	43 % 46 %
Low income Middle income Good income	33 % 42 % 57 %	31 % 38 % 50 %
Italians Non Italians	48 % 44 %	44 % 42 %
Educational level^a^ 1- 2 3–4 5–8	39 % 46 % 51 % 51 %	33 % 42 % 46 % 49 %

a Educational level is based upon the International standard classification of education (ISCED), 1997 version, and refers to: 1-pre-primary, primary education; 2-lower secondary education; 3–4-upper secondary and post-secondary non-tertiary education; 5–8 tertiary education.

We found that the most important factor influencing attendance to colorectal cancer screening was the level of educational attainment and financial income. Several differences exist between the economic structure of the Health System in USA and Italy. In Italy the Health System provides free care and screening for all population, in USA private insurances represent major payers for private care. Despite these differences, people with higher income had a statistically significant higher attendance rates to screening in Italy, probably related with associated higher educational attainment.

It is possible that education and information may increase the attendance to screening programs and to overcome the problem of differences related with race [3,4]. There is room for improvement. In Italy an invitation letter was sent only to 35 % of the population aged 50–69 years.

Even if there were statistically significant differences related with educational attainment and family income, still the overall attendance rate was less than 50 %. Proper education and information about the importance of preventive care may increase the attendance to screening programs [[7], [8], [9]]. Education and information in this setting should consider the cultural and social level of the public. A valid information requires several forms of communication including general and local specific considerations. The first form of communication should be a commitment from national institutions, including mass media campaigns, teaching in schools, in universities and in work places; the second aspect, probably the most difficult, should be reserved to local communities, including clinicians, nurses, small hospitals^.^. In this context a close collaboration between policy makers, health care providers and surgeons is fundamental, assuring a good cost-effectiveness ratio of health spending [10].

## Funding

No funds were received for this work.

## CRediT authorship contribution statement

**Raimondo Gabriele:** Data curation, Formal analysis, Validation, Visualization, Writing – review & editing. **Monica Campagnol:** Data curation, Formal analysis, Investigation, Methodology, Validation, Visualization, Writing – review & editing. **Immacolata Iannone:** Data curation, Formal analysis, Resources, Software, Validation, Visualization, Writing – review & editing. **Valeria Borrelli:** Data curation, Software, Supervision, Validation, Visualization, Writing – review & editing. **Antonio V. Sterpetti:** Conceptualization, Supervision, Validation, Visualization, Writing – original draft.

## Declaration of competing interest

The authors declare that they have no known competing financial interests or personal relationships that could have appeared to influence the work reported in this paper.
